# Mutation in the 26S proteasome regulatory subunit *rpn2* gene in *Plasmodium falciparum* confers resistance to artemisinin

**DOI:** 10.3389/fcimb.2024.1342856

**Published:** 2024-02-09

**Authors:** Adriana F. Gonçalves, Ana Lima-Pinheiro, Miguel Teixeira, Gustavo Capatti Cassiano, Pedro Cravo, Pedro E. Ferreira

**Affiliations:** ^1^ Life and Health Sciences Research Institute (ICVS), School of Medicine, University of Minho, Braga, Portugal; ^2^ Life and Health Sciences Research Institute (ICVS)/ Biomaterials, Biodegradables and Biomimetics Research Group (3B's)-PT Government Associate Laboratory, Braga, Portugal; ^3^ Department of Protection of Specific Crops, InnovPlantProtect Collaborative Laboratory, Elvas, Portugal; ^4^ Global Health and Tropical Medicine (GHTM), Associate Laboratory in Translation and Innovation Towards Global Health (LA-REAL), Instituto de Higiene e Medicina Tropical (IHMT), Universidade Nova de Lisboa (UNL), Lisbon, Portugal

**Keywords:** malaria, *Plasmodium falciparum*, drug resistance, proteasome, genetic engineering, artemisinin

## Abstract

**Introduction:**

Malaria parasites increasingly develop resistance to all drugs available in the market, hampering the goal of reducing malaria burden.

**Methods:**

Herein, we evaluated the impact of a single-nucleotide variant, E738K, present in the 26S proteasome regulatory subunit *rpn2* gene, identified in *Plasmodium chabaudi* resistant parasites. Plasmids carrying a functional *rpn2* interspecies chimeric gene with 5’ recombination region from *P. falciparum* and 3’ from *P. chabaudi* were constructed and transfected into Dd2 *P. falciparum* parasites.

**Results and discussion:**

The 738K variant parasite line presented increased parasite survival when subjected to dihydroartemisinin (DHA), as well as increased chymotrypsin-like activity and decreased accumulation of polyubiquitinated proteins. We thus conclude that the ubiquitin-proteasome pathway, including the 738K variant, play an important role in parasite response to DHA, being the first report of a mutation in a potential DHA drug target enhancing parasite survival and contributing to a significant advance in the understanding the biology of artemisinin resistance.

## 1 Introduction

Decreasing the burden of malaria and related fatalities has been hampered by malaria parasites’ ability to evolve resistance to all treatments available on the market (Antony and Parija, 2016). Therefore, understanding the mechanisms by which parasites acquire resistance to antimalarials is crucial for the future development of alternative effective treatments. Nowadays, artemisinin and its derivatives (ARTs) are the recommended course of treatment, together with a long-term partner, forming the Artemisinin-based combination therapies (ACTs). Artemisinin resistance, defined mostly by ring-stage survival assays (RSA), has been frequently associated with mutations in the K13 protein, which do not modulate proteasome activity (Wicht et al., 2020). However, using a proteasome inhibitor, such as epoxomicin, increases artemisinin activity in resistant and sensitive parasites (Bozdech et al., 2015). On that account, mutations in different parts of the ubiquitin-proteasome pathway (UPP) may impact artemisinin responses (Bridgford et al., 2018). Recent studies demonstrated that mutations in the 19S and 20S proteasome subunits sensitized K13^C580Y^ parasites, the most prevalent artemisinin resistance-associated mutation in the Greater Mekong Subregion, based on RSA (Rosenthal and Ng, 2021; Rosenthal and Ng, 2023). Additionally, two mutations in a gene encoding a deubiquitinating enzyme, UBP-1, were identified in artemisinin resistant *P. chabaudi* parasites and it was shown that they can mediate ARTs resistance in *P. falciparum* (Cravo, 2022).

The ubiquitin-proteasome system is essential to eukaryotic cells as it is responsible for the degradation or recycling of proteins, influencing several cellular processes, including cell cycle, transcriptional regulation, cellular stress response, signal transduction, and cellular trafficking (Wang et al., 2015). This protein regulation is critical for the rapid transformations that malaria parasites undergo during the life cycle progression between the two hosts, especially in the stages with high replication rates (Krishnan and Williamson, 2018). The UPP involves a process of protein posttranslational modifications, called ubiquitination, which attaches polyubiquitin chains to proteins that are subsequently recognized by the 26S proteasome. The type of ubiquitination defines if the protein is recycled or degraded by the proteasome (Aminake et al., 2012; Wang et al., 2015).

The 26S proteasome, a barrel-shaped multi-subunit proteinase complex, is divided into a 20S core particle (CP) and a 19S regulatory particle (RP). The latter is responsible for the recognition, deubiquitination, unfolding, and translocation of substrates. The 20S core is responsible for proteolysis via peptidylglutamyl-peptide hydrolytic (PGDH) (caspase-like), trypsin-like, and chymotrypsin-like activities, encountered in three b-subunits (b1, b2, and b5, respectively) (Wang et al., 2015). These catalytically active subunits use an N-terminal threonine as the nucleophile and cleave after the carboxy-terminal side of acidic, tryptic, and hydrophobic residues, respectively. These active sites are located in the two inner beta rings of the barrel, consisting of b1-b7, while the two outer rings consist of a1-a7, which prevent polypeptide access to the catalytic subunits. This gate opening of the 20S core is regulated by the 19S subunits (Li et al., 2016; Ng et al., 2017).

The 19S RP is structurally divided into two subcomplexes, lid and base. The lid subcomplex comprises nine regulatory particle non-ATPase (rpn) subunits (3, 5, 6, 7, 8, 9, 11, 12, 15), while the base subcomplex includes the other rpn and six paralogous distinct regulatory particle ATPase (rpt) subunits. These latter are responsible for the protein unfolding and the CP gate opening. In addition, they also stabilize the interaction of the 19S RP with the 20S CP (Mao, 2021). The ubiquitin receptors within the base, including rpn1, rpn2, rpn10, and rpn13, are responsible for recognizing the ubiquitylated substrate, which is the first step of substrate processing by the proteasome. Rpn1 and rpn2 are the two largest proteasome subunits and function as scaffolds and coordinate the activity and placement of multiple ubiquitin-processing factors at the proteasome (Rosenzweig et al., 2012; Mao, 2021).

To understand the role of the UPP in the artemisinin response and resistance, we studied the impact of the E738K single nucleotide variant (SNV), previously identified in the 26S proteasome regulatory subunit *rpn2* gene of *P. chabaudi* parasites resistant to artesunate + mefloquine through Whole Genome sequencing (WGS) (Rodrigues and Cravo, 2010). Herein, we demonstrated that the variant influences parasite survival and proteasome activity when subjected to artemisinin treatment demonstrating the importance of the UPP in the parasite and towards artemisinin response.

## 2 Materials and methods

### 2.1 Cloning strategy

DNA sequences were obtained using the database PlasmoDB (www.plasmodb.org). *P. chabaudi* gene ID is PCHAS_133430 and *P. falciparum* is PfDd2_140070600. Cloning strategies (**Supplementary Figure 1**) were designed in silico with the software Ape.

#### 2.1.1 *P. falciparum* and *P. chabaudi* gDNA extraction

*P. falciparum* genomic DNA (gDNA) was extracted from a Dd2 parasite culture, while *P. chabaudi* gDNA was obtained from a blood sample of infected mice from the collaborator Dr. Pedro Cravo from Global Health and Tropical Medicine (GHTM), Instituto de Higiene e Medicina Tropical (IHMT), Universidade Nova de Lisboa. Both samples were extracted using the NZY Blood gDNA isolation (NZYTech). Concentration and purity were measured using a NanoDrop™1000 spectrophotometer (Thermo Scientific), and the gDNA was stored at -20°C.

#### 2.1.2 Construction of *Plasmodium* spp. *rpn2* chimeric gene

To construct a *Plasmodium* spp. interspecies chimeric gene, Selection-Linked Integration (SLI) technique was used (Birnbaum et al., 2017). To mitigate potential structural constraints, genetic regions of interest were chosen according to the protein’s 3D model. The Swiss Model automated protein structure homology-modelling server was employed to generate this 3D model, using the structure of the 26S proteasome regulatory subunit rpn2 from *Plasmodium falciparum* (isolate 3D7) with the repository database code Q8IKH3 (Q8IKH3_PLAF7) (https://swissmodel.expasy.org/repository)”.

Then, the regions of interest of each species were amplified through a Polymerase Chain Reaction (PCR). Primers were designed considering the gene sequence, being the reverse primer from *P. falciparum* (p2 – **Table 1**) and the forward primer from *P. chabaudi* (p3 – **Table 1**) complementary allowing subsequent fusion. Samples were analyzed by electrophoresis in an agarose gel (1%), using a molecular weight marker (GeneRuler 1kb DNA Ladder, Thermo Fisher Scientific) to confirm that the obtained fragments had the expected size.

**Table 1:** Primer sequences used for plasmid construction and genotyping.

Name	Primer Sequence (5’^®^3’)	Tm
p1	ACTCGCGGCCGCTAAGAAAATGAAAGTAATGCAATAACGA	69°C
p2	TATCATGTGCATATGCTAGCAATTCGTCTATACATTTATC	62°C
p3	TATAGACGAATTGCTAGCATATGCaCATGATACACAACAT	64°C
p4	TTTGAGCCCTTTACTTGGAAAGATGAAAACGTCGACGGAG	68°C
p5	GATACACAACATGAAAAAATAACAAGAGCATGTAGTATA	60°C
p6	GTTATTTTTTCATGTTGTGTATCATGTGCATATGCTAGC	62°C
p7	TATCGTTACGTCCGCCAGTG	58°C
p8	CTCCTGCACAAGTTCCAATG	55°C

A second PCR was performed to fuse both fragments using the forward primer of the *P. falciparum* part (p1 – **Table 1**) and the reverse primer of the *P. chabaudi* part (p4 – **Table 1**) with iProof HF DNA Polymerase (Bio-Rad Laboratories). Expected PCR band size was extracted using GRS PCR & Gel Band Purification kit (GRiSP, Lda) and sequenced (STAB Vida).

#### 2.1.3 Genetic engineering of *rpn2* 738K variant

The previous product was posteriorly inserted in the JET plasmid (CloneJET PCR Cloning kit, Thermo Fisher Scientific) applying the manufacturer´s protocol. Reaction products were then transformed into chemically competent Escherichia coli cells (XL-10) by heat shock. Posteriorly, cells were transfered to lysogeny broth (LB) medium [10 g/L bacto-tryptone, 5 g/L yeast extract, and 10 g/L NaCl] plates supplemented with ampicillin antibiotic (100 µg/mL) and incubated overnight at 37°C. The next day, two of the emerged colonies were grown and plasmid DNA was extracted using the Zyppy™ Plasmid Miniprep Kit (Zymo Research). Concentration and purity were measured using a NanoDrop™1000 spectrophotometer (Thermo Scientific). Finally, to confirm the DNA sequence of the construct, sequencing was carried out by STAB Vida.

The plasmid with the 738K variant was constructed using the Selection-linked Integration (SLI) technique (Birnbaum et al., 2017). This method makes it possible to obtain parasite lines with genomic integrations in *P. falciparum* with a greater speed and success rate. Therefore, the previous fragment was inserted in the Selection-Linked Integration plasmid (pSLI), being first needed to digest both the pSLI ((Plasmid #85791) - Addgene) and the fragment with NotI and SalI (New England Biolabs). Electrophoresis was used to verify the digestion, and the desired bands were extracted with the GRS PCR & Gel Band Purification kit (GRiSP, Lda). Then, ligation of the fragment to the plasmid was carried out using T4 DNA Ligase (Thermo Fisher Scientific). The ligation product was transformed into competent *E. coli* cells and, posteriorly, the plasmid was extracted using the NZYMiniprep kit (NZYTech, Lda.).

#### 2.1.4 Genetic engineering of *rpn2* E738 variant

A descendent plasmid was then constructed with the E738 variant using the site-directed mutagenesis technique to replace a lysine for a glutamic acid on the 738 position (Liu and Naismith, 2008). Using primers p5 and p6 (**Table 1**), a PCR was performed. The amplified product was then digested with DpnI endonuclease (New England Biolabs) at 37°C for 3 hours to select the mutation-containing synthesized DNA, digesting the parental DNA template. The mutated plasmid was then transformed into chemically competent *E. coli*. Posteriorly, expanded selected colonies were extracted using the Zyppy™ Plasmid Miniprep Kit (Zymo Research). After plasmid DNA extraction, concentration and purity were measured using a NanoDrop™ 1000 spectrophotometer (Thermo Scientific) and sent for sequencing at STAB Vida.

### 2.2 Transgene *P. falciparum* cell lines

#### 2.2.1 Transfection

Both plasmids were then transfected into Dd2 *P. falciparum* parasites cell lines, a multi-drug resistant strain derived from an Indochina isolate (Reilly et al., 2007). Transfection was performed by electroporation in uninfected erythrocytes (Hasenkamp et al., 2012). First, erythrocytes are washed with cytomix [10 mM/L K^2^HPO^4^/KH^2^PO^4^, 120 mM/L KCl, 0.15 mM/L CaCl_2_, 5 mM/L MgCl_2_, 25 mM/L HEPES, 2 mM/L EGTA, adjusted with 10 M/L KOH to pH 7.6]. Afterward, 300 µL of erythrocytes were added to the plasmid solution, which contains 50 µg of plasmid and up to 200 µL of cytomix. This was transferred to a Gene Pulser/MicroPulser™ Electroporation Cuvettes, 0.2 cm gap (Bio-Rad Laboratories, Inc) and eletric shocked on the Gene Pulser Xcell™ (Bio-Rad Laboratories) electroporator. The mixture was washed twice in 5 mL of malaria culture medium (MCM) [RPMI, 1640 (Gibco) with 2 mM L-glutamine, 200 µM hypoxanthine, 0.25 µg/mL gentamycin, 25 mM HEPES, 0.2% NaHCO3, and 0.25% Albumax II (Life Technologies)] to remove lysed erythrocytes. The transfected erythrocytes were then inoculated with *P. falciparum* Dd2 strain parasite erythrocytes at the trophozoite stage and 5 mL of MCM.

#### 2.2.2 *P. falciparum* culture

Parasite cultures were maintained under a controlled atmosphere of 5% O_2_/5% CO_2_/90% N2 at 37°C in a CO_2_ incubator (Thermo Fischer Scientific). Cultures on a t25 flask were maintained at 4% hematocrit in 5 mL of MCM. Medium was changed every other day, and cultures’ healthiness and parasitemia were regularly monitored by microscopy. This microscopic analysis was made using a blood smear, fixed with methanol (100%), and colored with 10% Giemsa’s eosin methylene blue solution (Merck) for 20 min. Parasitemia was calculated by dividing the number of parasitized erythrocytes per total counted erythrocytes.

#### 2.2.3 Parasite transgenic lines drug selection

To select the genetically modified parasites, first, the parasites with episomal pSLI were selected with WR99210, followed by a second selection with neomycin (G418), which allows the selection of parasites with the plasmid genome integrated, since a promoterless resistance cassette is present in it.

#### 2.2.4 Genotyping

After the *P. falciparum* drug selection and culture growth, parasites were genotyped and evaluated for successful genetic manipulation. For that, gDNA was extracted from parasite cultures using the NZY Blood gDNA Isolation kit (NZYTech). To perform the genotyping, a PCR was performed using the forward primer (p7 - **Table 1**) located in the *P. falciparum* region and the reverse primer (p8 - **Table 1**) in the *P. chabaudi* region, forming a band of, 2370 bp, being posteriorly digested with the restriction enzyme NheI (New England Biolabs). Then, DNA sequencing was carried out.

### 2.3 Phenotyping

#### 2.3.1 Parasite growth analysis

*P. falciparum* growth was evaluated using a fluorometric assay. For that, a parasite culture with ring-stage parasites starting at 0.5% parasitemia and 4% hematocrit was prepared. Throughout 14 days, 100 µL of culture was retrieved every day to a 96-well plate and stored at -20°C. Culture’s health was daily evaluated by blood smear.

To carry out the fluorimeter readings, plates were stained with SYBR Green. For this, 4 µM of SYBR green were added to the lysis buffer (15.76 g Tris-HCl, adjust pH to 7.5, 2% w/v EDTA, 0.016% w/v saponin, 1.6% v/v Triton X-100). In a new 96-well plate, 25 µL of the lysis buffer + SYBR Green mixture were added to each well. Afterward, 50 µL of the homogeneized cultures from the assay plates were added. Then, after an incubation of 3 hours at RT on a dark chamber, plates were analyzed in the fluorimeter (Thermo Scientific Varioskan Flash) with excitation and emission wavelength bands at 485 and 530 nm, respectively.

#### 2.3.2 *P. falciparum* schizogony analysis

The number of merozoites per schizont analysis was performed using a thin film Giemsa stain as described. Firstly, parasites were tightly synchronized using sorbitol (5%) into ring-stage. Then, after approximately 36 hours, the parasite stage was evaluated by a blood smear. Mature schizonts were observed, and merozoites were counted and normalized per number of schizonts.

#### 2.3.3 Half maximal inhibitory concentration (IC_50_)

Tightly synchronized ring-stage parasites at 0.5% parasitemia and 2% hematocrit were added to the plate previously prepared with several dilutions of DHA, 100 µL of the mixture (iRBCs, RBCs, and MCM) to each well. Plates were then incubated for 72 hours in the CO_2_ incubator. After the incubation, plates were sealed and frozen at -20°C. Subsequently, fluorimeter readings were executed as previously mentioned. IC_50_ values were calculated by nonlinear regression analysis using Graphpad^®^.

#### 2.3.4 *P. falciparum* survival assay

To perform a ring survival assay (RSA) and a trophozoite-stage survival assay, tightly synchronized early ring-stage and trophozoite-stage parasites, respectively, at 0.5% parasitemia and 2% hematocrit, were exposed to 2.5, 5, 10, 20, 50, 250, and 700 nm of DHA. After 6 hours of incubation in a CO_2_ incubator, parasites were centrifuged for 5 min at, 1500 xg, the supernatant was removed and washed three times with phosphate-buffered saline 1x (PBS). Finally, 1 mL of MCM was added to each pit and incubated for 66 hours. Plates were then stored at -20°C. Fluorimeter readings were carried out as previously mentioned. Survival (%) represents the parasitemia normalized to untreated (100%) survival and kill-treated (0% survival) controls, where kill-treated refers to samples treated with 700 nm DHA.

#### 2.3.5 Proteasome activity analysis

To analyze the proteasome activity, trophozoite-stage parasite cultures at 3% hematocrit and 8% parasitemia were first washed once with PBS (1x) and, consequently, incubated with 0.05% (w/v) saponin for 10 min on ice. Then, parasite pellets were washed with ice-cold PBS (1x) three times and lysed at -20°C overnight. It was then cleared by centrifugation at, 1000 xg for 10 min.

Cell lysates were quantified using the Bradford method. To perform the quantification, a 96-well plate was used, and 10 µL of the sample was mixed with 200 µL of 1x Bradford’s reagent (Bio-Rad Laboratories, Inc). After a 5 min incubation, the absorbances were read at 590 nm. Then, the concentration was determined using a calibration line for bovine serum albumin (BSA) concentration absorbances, which started with a, 2000 µg/mL concentration and continued with dilutions of 1:2.

The assay was then performed using the Proteasome Activity Fluorometric Assay Kit II (Ubiquitin-Proteasome Biotechnologies) and following its recommendations. For that, in a 96-well plate, 10 µL of cell lysate was mixed with 20x proteasome assay buffer and 0, 3.125, 6.25, 12.5, and 25 µM of DHA, for a total volume of 50 µL and incubated for 30 min at 37°C. Meanwhile, the proteasome substrate was prepared, mixing 50 µL of 20x proteasome assay buffer with 950 µL H_2_O, incubating for 10 min at 37°C and, posteriorly, adding 2 µL of, 1000x Suc-LLVY-AMC substrate (50 mM in DMSO (dimethyl sulfoxide)) to measure the chymotrypsin-like activity, and stored at 37°C after vortexing for 10 seconds. After the 30 min incubation, 50 µL of proteasome substrate was added to each pit. Fluorescence was measured at 360/40 nm and 460/30 nm for excitation and emission, respectively, on the spectrophotometer (Thermo Scientific Varioskan Flash), previously heated at 37°C, with readings at every 5 min for 3 hours. For proteasome activity analysis, the slope value of each curve was calculated between 20 and 90 min by using the formula (Y90-Y20)/(X90-X20), in which Y90 and Y20 were AMC fluorescence readings from the Y-axis at 90 min and 20 min, respectively. Values are normalized to the untreated sample.

#### 2.3.6 Ubiquitination activity assay

A Western Blot was used to evaluate the differences in the levels of ubiquitinated proteins. For that, trophozoite-stage parasites were first incubated with 5 mL of MCM treated with 0, 0.01, 0.1, 1, 10 µM of DHA for 90 min at 37°C with a controlled atmosphere. Then, trophozoites were isolated using the AutoMacs^®^ Pro Separator (Miltenyi Biotec), separating different stage-parasites using magnetic means. After separation, the eluate was centrifuged at, 1500 xg for 5 min and washed 3 times with PBS 1x, forming a dark pellet with the parasites. The supernatant was discarded, and 50 µL of H_2_O was added, storing at -20°C to lyse the parasites. Later on, lysates were cleared by centrifugation for 15 min at, 5000 xg at 4°C and supernatant was collected. Protein quantification was assessed using the Bradford method previously described.

Electrophoresis was performed using a running gel of 12% sodium dodecyl sulfate-polyacrylamide gel electrophoresis (SDS-PAGE) and a stacking gel of 5% SDS-PAGE. Protein samples were prepared by adding 20 µg of total protein to 10 µL of loading buffer, previously prepared by adding 50 µL of 2-mercaptoethanol to 950 µL of 2x Laemmli Sample Buffer (Bio-Rad Laboratories). Next, proteins were denaturated for 5 min at 98°C. The samples were loaded onto the gel and separated by electrophoresis (100V). Gels were then transferred to a nitrocellulose membrane (Bio-Rad Laboratories) in a Trans-Blot Turbo Transfer Buffer (Bio-Rad Laboratories) using the Trans-Blot Turbo Transfer System (Bio-Rad Laboratories). To prevent unspecific connections, the membranes were blocked with 5% of BSA in Tris-Buffered Saline (TBS)/0,1% Tween 20 for 1h at RT and then incubated overnight, at 4°C, with the primary antibodies: ubiquitin (1:1000, source: rabbit, Invitrogen) and actin (1:1000, source: mouse, Invitrogen). Actin was used as the loading control. On the next day, the membranes were washed in TBS/0,1% tween three times, each of 5 min, and then incubated for 1 hour with fluorescent secondary antibodies, respectively: anti-rabbit (1:1000, source: goat, conjugate: DyLight 488, Invitrogen) and anti-mouse (1:1000, source: goat, conjugate: DyLight 680, Invitrogen). Sapphire Biomolecular Imager (Azure Biosystems) was used for protein detection. ImageJ software was used for quantitative analyses.

#### 2.3.7 Statistical analysis

Statistical analyzes were performed using the GraphPad Prism 8 software. Results were presented as means ± standard error of the mean (SEM). Comparisons between different conditions were made using t-tests. Differences were considered significant in the statistical analysis when *p*<0.05. At least three biological replicates were performed for each assay.

## 3 Results and discussion

### 3.1 Transgene *P. falciparum* parasite lines

**Figure 1:**
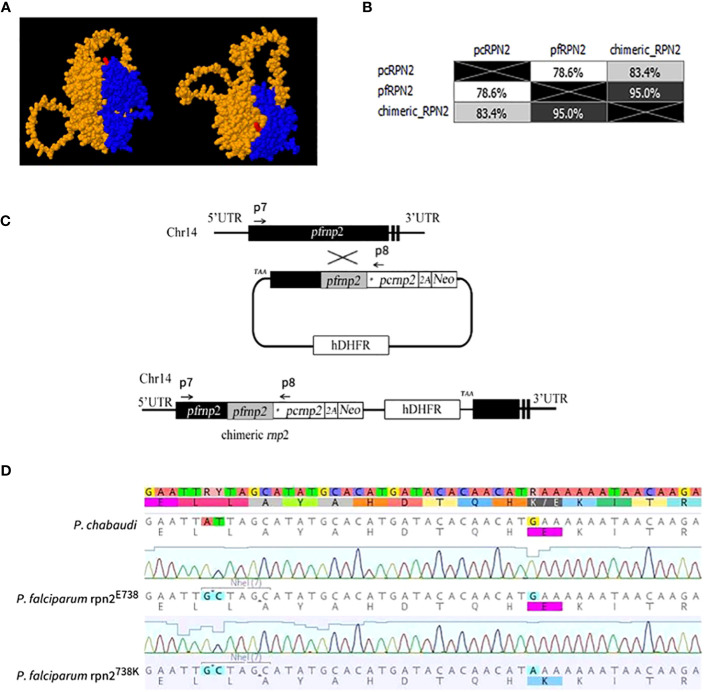
Strategy and plasmid construction for 738K variant. **(A)**. Localization of the 738K mutation in a protein 3D model of the interspecies chimeric rpn2 protein. The blue region corresponds to the *P. falciparum* carboxylic terminal and the orange to the amino terminal of the *P. chabaudi* protein sequence. The E738K variant location is indicated in red. **(B)**. Identity matrix of the results from the protein sequence BLOSUM95 alignment between *P. chabaudi*, *P. falciparum*, and the chimeric RPN2 hybrid. **(C)**. Strategy to construct a plasmid with the 738K variant. Using the Selection-Linked Integration (SLI) technique, a plasmid with 5’ recombination region from *P. falciparum* and 3’ from *P. chabaudi*, where the E738K variant is located (marked with an asterix). **(D)**. PCR sequencing for confirmation of synonymous mutation engineering for the NheI restriction site and non-synonymous E738K variants in *P. chabaudi*, *P. falciparum* rpn2^E738^ and *P. falciparum* rpn2^738K^ parasite lines.

The genome of ACT-resistant *P. chabaudi* parasites was investigated previously for artemisinin resistance-associated mutations (Rodrigues and Cravo, 2010; Rodrigues et al., 2010; Borges et al., 2011; Martinelli et al., 2011). As a result, a SNV in the 26S proteasome regulatory subunit *rpn2* gene, causing an E738K substitution was identified and suggested to alter protein network interactions in the resistant parasites. Therefore, to evaluate the impact of this SNV in the proteasome function and artemisinin response in *P. falciparum* and taking into account the protein 3D protein structure (**Figure 1A**), plasmids were constructed carrying a functional *rpn2* chimeric gene (Cravo, 2022; Mok et al., 2023). Protein sequence alignment show 78.6% identity between the *rpn2* gene of *P. chabaudi* and *P. falciparum* (**Figure 1B** and **Supplementary Figure 2**). The E738K variant was studied generating the rpn2^E738^ and rpn2^738K^ plasmids, as shown in the workflow scheme (**Figure 1C**).

To assess the correctness of the transfected cultures, these were genotyped by PCR, and the reaction products were sequenced to confirm the different variants (**Figure 1D**). The sequencing electropherogram showed that the E738 variant is present in the *rpn2* gene sequence of *P. chabaudi* (PCHAS_133430), and no restriction site for the NheI enzyme is present. On the other hand, in the mutated *P. falciparum* parasite lines, a restriction site for NheI is observed, as well as both variants, E738, and 738K, in the different lines, confirming the successful genetic engineering of the parasite lines.

### 3.2 Parasite growth analysis

**Figure 2:**
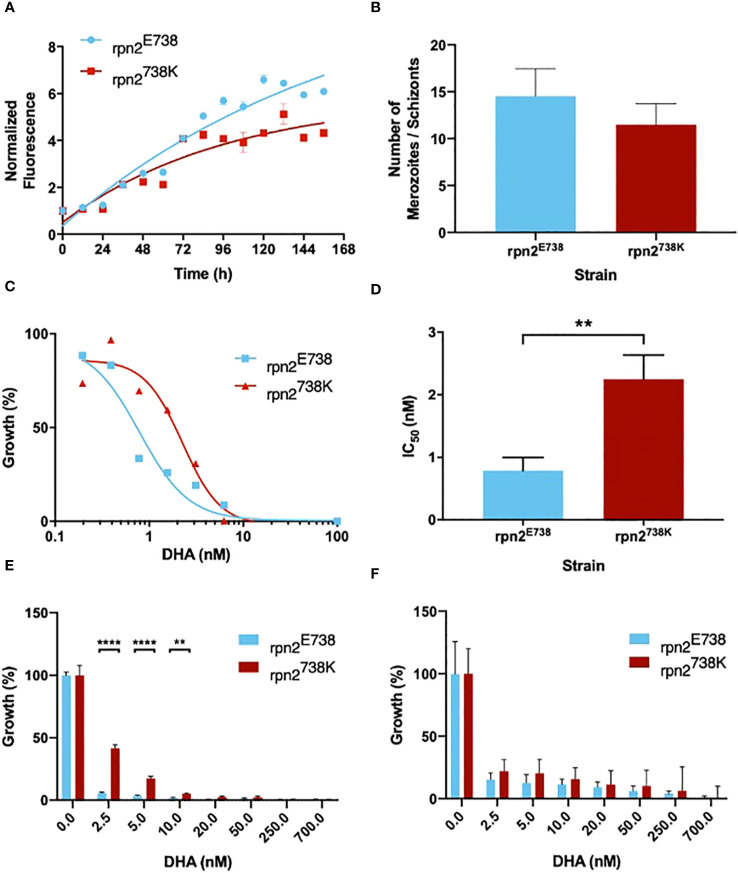
rpn2738K influences parasite growth and susceptibility to DHA. **(A)**. Growth curves of parasite lines rpn2E738 and rpn2738K. Parasitemia was evaluated every day for 14 days. **(B)**. Mean ± SEM of merozoites number within schizonts are represented for parasite lines rpn2E738 and rpn2738K. **(C)**. *In vitro* IC_50_ of rpn2E738 and rpn2738K. Ring-Stage parasites were exposed to a range of DHA concentrations for 72 hours. Dose-response curves are plotted from mean % inhibition ± SEM. **(D)**. Mean ± SEM IC_50_ values are represented for dihydroartemisinin. Three assays were performed for each line. Statistical evaluations comparing both lines were performed using unpaired *t*-tests. **(E)**. Young rings (0-3h post-invasion) of the rpn2E738 and rpn2738K lines, were treated with a pulse of DHA for 6h. Ring survival rate ± SEM were measured. **(F)**. Trophozoite-stage parasites of the rpn2E738 and rpn2738K were subjected to the treatment mentioned before and survival rate ± SEM were measured. Three assays were performed for each line. Statistical evaluations comparing lines were performed using two-way ANOVA. **p<0.01, ****p<0.0001.

A different growth pattern was observed during the maintenance of the two cultures with the variants. To understand these differences, their growth was analyzed with parasitemia counts every day for 14 days. With this experiment, it was possible to perceive that the 738K variant grew slower than its E738 counterpart (**Figure 2A**). To understand if this growth difference was due to metabolic differences or cellular multiplication alteration, the number of merozoites was counted for both parasite lines. It was observed that rpn2^E738^ parasites presented a greater number of merozoites per schizont, with an average of 14, compared to rpn2^738K^ parasites, with an average of approximately 12 merozoites. However, this difference was not statistically significant (**Figure 2B**). In the Dd2 strain, the range of 8 to 28 merozoites are usually counted, with an average of 18 merozoites per schizont (Simon et al., 2021).

At the end of schizogony, cytokinesis results in the development of new merozoites, which are then released by rupture of the host erythrocyte. During schizogony, cellular structures such as secretory organelles, rhoptries, and dense granules are generated. Parasites also modify their host cell, secreting several proteins into the cytoplasm and affecting the erythrocyte’s cytoskeleton and surface. Merozoite’s secretory organelles are dismantled after the invasion, increasing protein ubiquitylation, since protein degradation processes are essential for the asexual intraerythrocytic cycle. In addition, inhibition of the UPP results in the reduction of parasite infection (Green et al., 2020; Rashidi et al., 2021). Accordingly, the 738K variant may have an influence on proteasome function in the schizogony stage, resulting in slower growth and lower parasitemia.

### 3.3 Susceptibility assays

#### 3.3.1 Half maximal inhibitory concentration (IC_50_)

To assess the impact of the E738K variant on the response to DHA, *in vitro* drug susceptibility assays were performed. The number of live parasites previously exposed to serial dilutions of DHA was quantified through fluorimeter readings, and IC_50_s were calculated. The 738K variant led to a near four-fold significant increase in the IC_50_ compared to the E738 variant (**Figures 2C, D**), with values of 2.25 nM and 0.8 nM, respectively.

Typically, the IC_50_ values for DHA vary between 0 and 8 nM in *P. falciparum* (Fairhurst and Dondorp, 2016). Both parasite lines are within the strain IC_50_ interval usually observed. However, there is a significant difference regarding artemisinin response. Nevertheless, *in vivo* parasite clearance half-lives correlate poorly with DHA 50% inhibitory concentrations *in vitro*, therefore parasite survival assays was used to evaluate the difference in the artemisinin response (Fairhurst and Dondorp, 2016).

#### 3.3.2 Parasite survival assay

Artemisinin resistance was originally difficult to investigate due to poor correlation with the existing standard *in vitro* measures of susceptibility, which complicated the understanding of the biology behind the slow-clearing *P. falciparum* in Cambodia (Noedl et al., 2008; Fairhurst and Dondorp, 2016). It was later discovered that parasite resistance to ARTs did not emerge across the whole parasite life cycle, and it was only identifiable in the ring stage of development (Khoury et al., 2020). Given the need for a more reliable and standardized *in vitro* measure of delayed clearance that correlates with the *in vivo* resistance phenotype, the RSA was developed (Witkowski et al., 2013). This method consists of tightly synchronizing cultured parasites at the early ring stage and subjecting them to a 6-hour pulse of DHA. After 72 hours, the parasite survival rate is evaluated (Zhang et al., 2017; Sutherland et al., 2021).

RSA was performed to confirm the impact on the response to artemisinin. It was possible to observe a significant difference at 2.5, 5, and 10 nM of DHA between both lines, with the 738K variant demonstrating increased parasite survival (**Figure 2E**). The assay was also executed in trophozoites. In spite of the fact that rpn2^738K^ line present higher parasite survival, there was no significant difference between both lines at trophozoite stages (**Figure 2F**). This difference between the assay in rings and trophozoites corroborates that later parasite stages are highly susceptible to ART, unlike early ring-stage parasites that are able to survive pulses of DHA (Davis et al., 2020). This profile coincides with the peak period of hemoglobin uptake and degradation, during which Fe2+-heme is liberated and activated ART (Stokes et al., 2019).

#### 3.3.3 Evaluation of proteasome activity and ubiquitination

DHA reacts with susceptible groups in biomolecules in its activated state, causing cellular damage. These damaged proteins are subsequently marked with ubiquitin, rapidly increasing the accumulation of ubiquitinated proteins in DHA-treated parasites. This rapid accumulation may clog the proteasome and lead to the inhibition of its activity (Bridgford et al., 2018).

**Figure 3:**
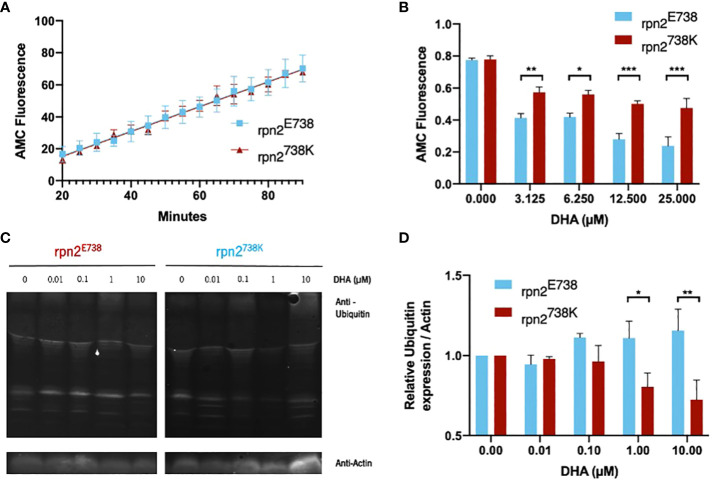
Impact of proteasomal activity and ubiquitination in rpn2^E738K^ *P. falciparum* cell lines. **(A)**. Chymotrypsin-like activity of trophozoite-stage parasites of the rpn2^E738^ and rpn2^738K^ lines without DHA was measured using the fluorogenic substrate Suc-LLVY-AMC for 3 hours. Slope value ± SEM was calculated using readings between 20- and 90-min. **(B)**. Trophozoite-stage parasites of the rpn2^E738^ and rpn2^738K^ lines, were incubated with DHA for 30 min and chymotrypsin-like activity was measured as previously described. Statistical evaluations comparing both lines were performed using a two-way ANOVA. **(C, D)**. Trophozoite-stage parasites of the rpn2^E738^ and rpn2^738K^ lines were incubated with DHA for 90 min, and protein was subsequently extracted to perform a western blot to analyze protein polyubiquitination. Relative ubiquitin expression considering actin expression. Ubiquitin values were normalized to the baseline (without DHA). Three assays were performed for each line in both essays. * *p*<0.05, **p<0.01, ***p<0.001.

Taking this into account, the chymotrypsin-like activity of the proteasome was analyzed with different DHA concentrations. In this essay, the proteasome cleaves the Suc-LLVY-AMC substrate, a fluorogenic substrate, while the released AMC fluorescence is monitored. In the baseline (without DHA), it was possible to observe that the rpn2^E738^ and rpn2^738K^ proteasome have similar activity (**Figure 3A**). When DHA was added, proteasome activity was inhibited in both lines, however the *P. falciparum* cell line with the 738K variant presented a significantly lower proteasome activity inhibition, with the difference increasing at the highest concentrations (**Figure 3B**).

In agreement with proteasome inhibition by DHA in *P. falciparum* (Bridgford et al., 2018), the accumulation of polyubiquitinated proteins increased, causing oxidative stress and inducing parasite death. The rpn2^738K^ parasite line presented a stabilization in the proteasome’s activity, which should have an impact at the level of polyubiquitinated protein accumulation. Therefore, a western blot experiment was performed to assess the level of polyubiquitinated proteins in DHA-treated parasites (**Figure 3C**). We observed an accumulation of polyubiquitinated proteins in the rpn2^E738^ parasites line compared with the baseline (without DHA). In the rpn2^738K^ parasite line, the relative ubiquitin presented a slight decrease at 0.1 µM concentration and was significantly smaller compared to the rpn2^E738^ parasites line at 1µM and 10µM concentrations (**Figure 3D**).

To further examine the proteasome activity in parasites treated with DHA, we tried to use transfectants expressing GFP coupled to a destabilization domain (DD), which is targeted for degradation by the proteasome in the absence of the protective ligand, Shield-1 (Bridgford et al., 2018). We were able to generate a parasite line carrying the E738 variant. However, after several attempts, we were unable to generate the parasite line bearing the 738K variant. We believe that the reason was possibly due to its slower growth and parasitemia characteristics, compared to the parasite line with the E738 variant. This difference in transfection capability was tested by attempting to transfect a plasmid with a GFP-only plasmid and similar result was observed.

DHA also disrupts proteasome-dependent degradation, preventing the removal of damaged proteins, which was observed with the accumulation of polyubiquitinated proteins. This is a double threat to parasites and further induces oxidative stress, leading to parasite death. Nevertheless, it was also shown that parasites with the 738K variant have less inhibition of the proteasome activity, as well as a consequent decrease in the accumulation of polyubiquitinated proteins. This suggests that this rpn2 variant antagonizes the physiological impact of DHA controlled by the 26S proteasome.

This mutation in the rpn2 subunit is a scaffold to ubiquitin-processing factors and is involved in stabilizing the 19S RP, responsible for the recognition of ubiquitylated substrates (Kors et al., 2019; Mao, 2021). A study in the human proteasome demonstrated that kinase p38 MAPK (Mitogen-Activated Protein Kinase) phosphorylated the rpn2 subunit, being Thr-273 of rpn2 the major phosphorylation site affected, stabilizing the poly- and non-ubiquitinated substrates and reducing all three proteolytic activities of the proteasome, caspase-, trypsin- and chymotrypsin-like activities. Additionally, this study also demonstrated that a T273A mutation in the rpn2 blocked the p38 MAPK-mediated proteasome inhibition. Other studies demonstrated that rpn2 interacts with rpt3, rpt4, and rpt6 of the proteasome ATPase complex, which are involved in regulating the 20S CP gate opening (Lee et al., 2010). Therefore, our results suggest that alterations in rpn2 protein have a major impact downstream of the 26S proteasome-mediated protein degradation process in *P. falciparum*.

## 4 Conclusion

Despite decades of clinical research and several treatment protocols, malaria is still responsible for an enormous global disease burden, with *P. falciparum* the primary species responsible for it. As the use and effectiveness of experimental vaccines have been residual, there is a significant reliance on antimalarial drugs both for prophylaxis and treatment of infected patients. Incomplete patient adherence coupled with extremely high treatment rates, among other factors have favored the evolution of drug-resistant *P. falciparum* mutants for many years (Wicht et al., 2020).

Artemisinin and its derivatives rapidly reduce parasite burden in *P. falciparum* infections. For this reason, nowadays, antimalarial control is highly dependent on ACTs, the combination of an ART with a longer-lasting partner drug. Nonetheless, decreased sensitivity to ARTs is emerging, making it critical to understand the mechanism of action of ARTs and their resistance (Bridgford et al., 2018).

The ubiquitin-proteasome system is a large and very complex proteomic network system. It is one of the main pathways responsible for regulating protein abundance levels and protein activity. Protein turnover, transcriptional regulation, cell cycle progression, differentiation, and signal transduction are some of the biological activities it plays a role (Hamilton et al., 2014). Additionally, it may also be involved in the mechanism of action of ARTs since these drugs damage parasite biomolecules, causing cellular damage and oxidative stress (Blasco et al., 2017). The finding of several mutations in different genes suggests that the evolution and spread of ART may occur in different manners. The 26S proteasome E738K in the rpn2 subunit substitution was originally identified through WGS of *P. chabaudi* ACT-resistant parasites that were generated by experimental evolution from ACT-sensitive progenitors (Rodrigues and Cravo, 2010). Protein sequence alignment showed 78.6% homology between the *P. falciparum* and *P. chabaudi* rpn2 subunits. Therefore, to study the E738K variant, two plasmids were manufactured with part of the *P. falciparum* gene and part of the *P. chabaudi* using selection-linked integration, where the variant is located, considering the protein 3D model. Subsequently, both plasmids were transfected into Dd2 *P. falciparum* parasites, creating the rpn2^E738^ and the rpn2^738K^ parasite lines. This approach shows a lot of potential for interspecies genetic studies for *Plasmodium* spp.

In the assays performed in these parasite lines, the line bearing the 738K variant presented increased IC_50_ values and significant differences in the parasite survival in the RSA. Regarding the proteasome activity, these parasites presented increased chymotrypsin-like activity when subjected to DHA and decreased accumulation of polyubiquitinated proteins.

Altogether, these results suggest that the 738K variant confers ARTs resistance through the reduction of cellular toxicity by 26S proteasome inhibition. Furthermore, proteasome activity assay and polyubiquitinated proteins assay, suggest that the proteasome has an important role in DHA resistance. Moreover, it demonstrates that the ubiquitin-proteasome pathway plays an important role in the mechanism of action of ARTs since there is increased proteasome activity and parasite survival in parasites treated with DHA.

Other yet unidentified mutations in this subunit of the proteasome may have an impact on the proteasome activity by modifying downstream phases of the degradation process. One hypothesis to understand the impact of the 738K variant in proteasome activity may be that DHA affetcts the phosphorylation of the glutamic acid present in the 738 position, which may be necessary to trigger proteasome inhibition. When the amino acid is changed to lysine in this position, similar to the T234A mutation (Lee et al., 2010), it may prevent proteasome inhibition. Accordingly, the amount of polyubiquitinated proteins does not increase until the point of oxidative stress and, consequently, parasite death.

In summary, this study has yielded novel insights into comprehending the proteasome’s involvement in DHA’s mechanism of action and resistance. Our findings substantiate the proposition that DHA might interact directly with the 26S proteasome in *P. falciparum*, marking the inaugural identification of a mutation in the *rpn*2 gene that modulates DHA resistance in *Plasmodium* spp.

However, future research must unravel the biological impact of the 738K variant for parasite growth and number of merozoites per schizont is crucial. Additionally, exploring this mutation in *Pfrpn2* gene and other naturally occurring mutations in *P. falciparum* warrants significant attention in upcoming investigations and development of artemisinin resistance.

## Data availability statement

The datasets presented in this study can be found in online repositories. The names of the repository/repositories and accession number(s) are the following: Plasmodium chabaudi chabaudi clone AS-ATN putative 26S proteasome regulatory subunit rpn2 mRNA, complete cds: https://www.ncbi.nlm.nih.gov/nuccore/2627951509 (GenBank: OR913732.1); Plasmodium spp. AG-2023a isolate E738 regulatory protein non-ATPase 2-like gene, complete sequence: https://www.ncbi.nlm.nih.gov/nuccore/OR833946 (GenBank: OR833946.1); Plasmodium spp. AG-2023a isolate 738K regulatory protein non-ATPase 2-like gene, complete sequence: https://www.ncbi.nlm.nih.gov/nuccore/OR833947 (GenBank: OR833947.1).

## Author contributions

AG: Formal Analysis, Investigation, Writing – original draft, Writing – review & editing. AL-P: Investigation, Writing – review & editing. MT: Investigation, Writing – review & editing. GC: Software, Writing – review & editing. PC: Methodology, Writing – review & editing. PF: Methodology, Writing – original draft, Writing – review & editing, Supervision.
