# Supplementary material for: Mutation in the 26S proteasome regulatory subunit rpn2 gene in Plasmodium falciparum confers resistance to artemisinin
Source: Front Cell Infect Microbiol. 2024 Feb 9;14:1342856. doi: 10.3389/fcimb.2024.1342856 (PMC10884193; doi:10.3389/fcimb.2024.1342856)

Supplementary Figure 1: Cloning strategy to obtain a *Plasmodium spp.* interspecies chimeric *rpn2* gene.

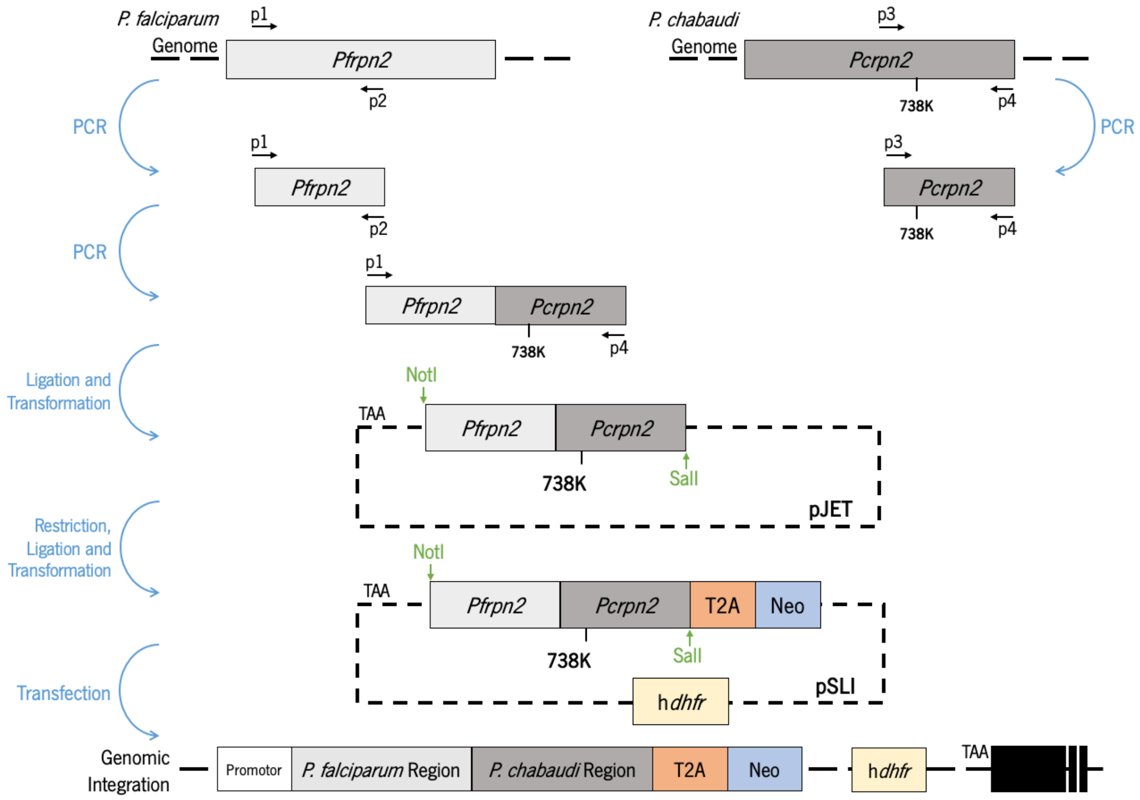

Supplement: Supplementary file 1 [file Image_1.pdf]
