# Supplementary material for: Mutation in the 26S proteasome regulatory subunit rpn2 gene in Plasmodium falciparum confers resistance to artemisinin
Source: Front Cell Infect Microbiol. 2024 Feb 9;14:1342856. doi: 10.3389/fcimb.2024.1342856 (PMC10884193; doi:10.3389/fcimb.2024.1342856)

Supplementary Figure 2: Protein sequence alignment between *P. chabaudi* rpn2, *P. falciparum* rpn2 and chimeric rpn2.

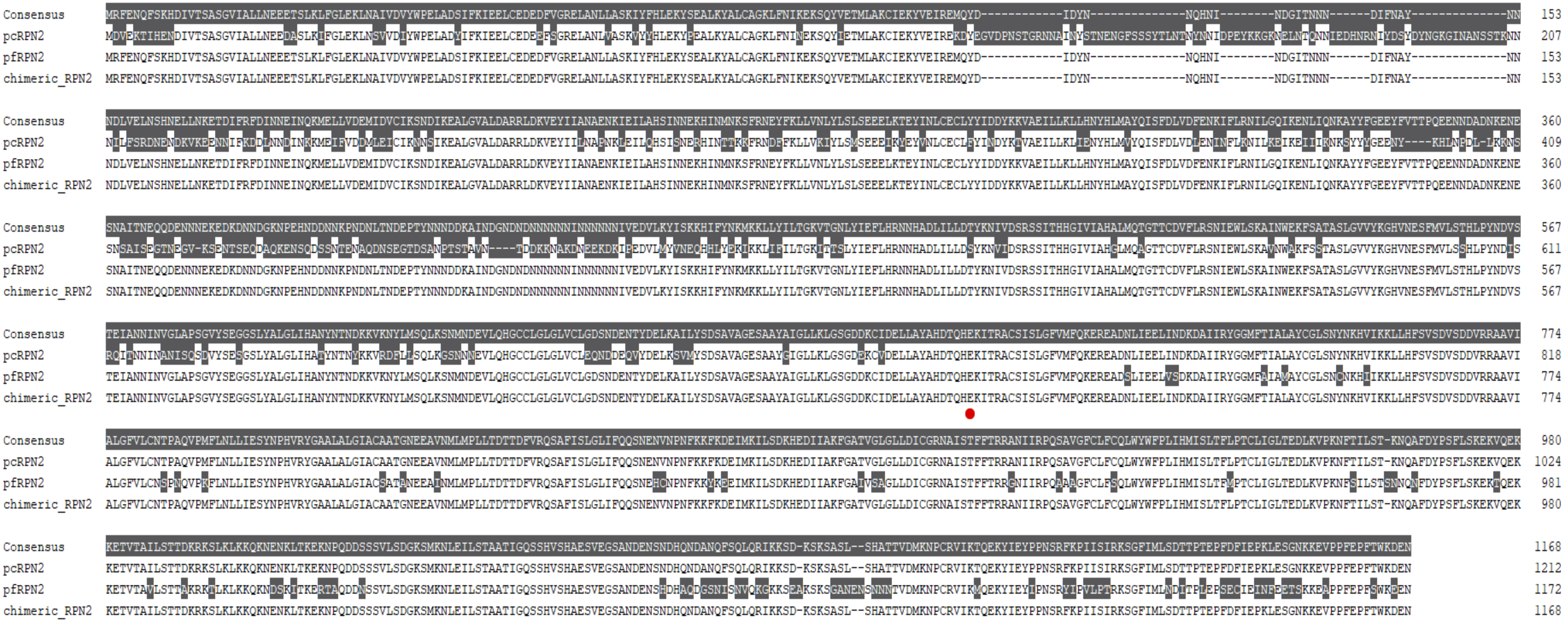

Supplement: Supplementary file 2 [file Image_2.pdf]
